# Combining Actigraph Link and PetPace Collar Data to Measure Activity, Proximity, and Physiological Responses in Freely Moving Dogs in a Natural Environment

**DOI:** 10.3390/ani8120230

**Published:** 2018-12-04

**Authors:** Heidi K. Ortmeyer, Lynda Robey, Tara McDonald

**Affiliations:** 1 Geriatric Research Education Clinical Center, VA Maryland Health Care System, Baltimore, MD 21201, USA; Lynda.Robey@va.gov (L.R.); Tara.McDonald@va.gov (T.M.); 2 Department of Medicine, University of Maryland School of Medicine, Baltimore, MD 21201, USA

**Keywords:** Actigraph, accelerometry, PetPace, proximity, rescue dogs, foster caretakers, pulse, respiration, heart rate variability, vasovagal tonal index

## Abstract

**Simple Summary:**

The Actigraph accelerometry monitors, the most widely used and extensively validated devices for measuring physical activity in humans, have also been validated for use in dogs. The ActiGraph GT9X Link monitor has Bluetooth Smart technology and a proximity-tagging feature that potentially allows for the measurement of distance between subjects, e.g., between human caretakers and their dog(s). The PetPace Smart-collar is a non-invasive wireless collar that collects important health markers, including heart beats, variation in the intervals between heartbeats, breaths per minute, and position data (lying, sitting, standing), in addition to activity. The purpose of this study was to determine whether combining data from the Actigraph monitor and PetPace collar would provide reliable pulse, respiration, and heart rate variability results during various activity levels in dogs, and whether these variables were affected by the absence or presence of their caretakers.

**Abstract:**

Although several studies have examined the effects of an owner’s absence and presence on a dog’s physiological responses under experimental conditions over short periods of time (minutes), little is known about the effects of proximity between humans and freely moving dogs under natural conditions over longer periods of time (days). The first aim of our study was to determine whether the combined data generated from the PetPace Collar and Actigraph Link accelerometer provide reliable pulse, respiration, and heart rate variability results during sedentary, light-moderate, and vigorous bouts in 11 freely moving dogs in a foster caretaker environment over 10–15 days. The second aim was to determine the effects of proximity (absence and presence of caretaker) and distance (caretaker and dog within 0–2 m) on the dogs’ physiological responses. Aim 1 results: Pulse and respiration were higher during light-moderate bouts compared to sedentary bouts, and higher at rest while the dogs were standing and sitting vs. lying. Heart rate variability (HRV) was not different between activity levels or position. Aim 2 results: During sedentary bouts, pulse and respiration were higher, and HRV lower, when there was a proximity signal (caretaker present) compared to no proximity signal (caretaker absent). Using multiple regression models, we found that activity, position, distance, and signal presence were predictors of physiological response in individual dogs during sedentary bouts. Our results suggest that combining data collected from Actigraph GT9X and PetPace monitors will provide useful information, both collectively and individually, on dogs’ physiological responses during activity, in various positions, and in proximity to their human caretaker.

## 1. Introduction

The Actigraph accelerometer (ActiGraph, Pensecola, FL, USA) was shown to be a valid, practical and reliable device for the measurement of habitual physical activity in dogs in 2011 [1]. The Actigraph monitor and other accelerometers have been used to assess activity in ideal weight, overweight and obese dogs [2], during weight loss in dogs [3], in dogs receiving chemotherapy [4], in shelter dogs [5], and to monitor the behavioral states in dogs [6]. ActiLife software (ActiGraph, Pensecola, FL, USA) allows researchers to process and score collected data via validated algorithms that include sedentary bout and activity bout analysis and physical activity intensity (cut-points). The newest models of the Actigraph accelerometer (wGT3X-BT and GT9X) have a proximity tagging feature, designed to evaluate the effect that location (or other subjects) have on a subject’s physical activity. Proximity tagging could be a useful tool in the study of human-animal interaction, allowing researchers to measure the effects of closeness/distance and presence/absence between humans and animals on behavioral and physiological responses in both species, over long periods of time (days to weeks). Although there are no published studies of using the proximity tagging features in dogs to date, a recent article examined the accuracy of proximity tagging to determine location in an office setting in 30 office workers [7].

The PetPace Smart-collar (PetPace, Burlington, MA, USA) is a non-invasive collar designed for dogs and cats that allows real-time, continuous, remote monitoring of activity, temperature, pulse, respiration, variations in heart rate (heart rate variability, HRV), and position (lying, sitting, standing). Data are uploaded to a cloud-based server, analyzed, and are available to owners and veterinarians through a subscription service. In the only published article on the PetPace collar to date, activity data collected from PetPace collars and Actigraph (GT3X) monitors were shown to be significantly related in 10 freely moving, active dogs over a period of seven days [8].

HRV, which is controlled by the autonomic nervous system, is the variation between successive heartbeats over a given period of time and is the result of the relative balances of parasympathetic and sympathetic input to the sinoatrial node. In a small sample size, Matooka et al. showed that interacting with an unknown friendly dog for two 30-min periods during six hours of continuous electrocardiograph monitoring increased HRV in older women; HRV was almost 2-fold higher in the presence of a dog than in the absence of a dog in the home [9]. The authors hypothesized that companion dogs buffer human stress and keep health problems at bay through increased parasympathetic neural capacity [9]. Indeed, a recent meta-analysis study has concluded that HRV is impacted by stress, and that the use of HRV as an objective assessment of physiological health and stress in humans is warranted [10]. 

HRV is an established risk factor for mortality in healthy dogs and in dogs with heart failure [11]. Whether and under what circumstances HRV can be utilized as an indicator of stress in dogs has not been well established. Studies performed in family dogs outside of the home showed HRV decreased during petting by a family member and increased when left alone [12]. Similarly, in a laboratory setting, HRV was higher in family dogs when the owner was not present vs. when the owner was present [13]. In both studies the measurements were obtained over a very short period of time (minutes). Whether HRV is affected by proximity between companion dogs and human caretakers in a family setting over a longer period of time (days) is not known. 

The first aim of our study was to establish whether combining data generated from the PetPace Collar and Actigraph Link accelerometer would provide reliable pulse, respiration, and heart rate variability results during sedentary, light-moderate, and vigorous bouts in 11 freely moving dogs in a foster caretaker environment over 10–15 days. The second aim was to determine the effects of proximity (absence and presence of caretakers) and distance (caretakers and dog within 0–2 m) on dogs’ physiological responses.

## 2. Materials and Methods

All dogs (*n* = 11) were American Eskimos and were under the care of experienced volunteer rescue group (Eskie Rescuers United American Eskimo Dog Rescue, Inc.) foster caretakers during the course of the study. Seven dogs had adult female foster caretakers and four dogs had adult male and female caretakers. There were no children living in the foster homes during the study. Study procedures were approved by the University of Maryland Institutional Research Board (HP-00074763) and Institutional Animal Care and Use Committee (HKO-061701A), and the Veterans Affairs Research & Development Committee (1200930).

Human and dog participants wore Bluetooth-enabled Actigraph GT9X Link accelerometers for 24 h/day (except 2–3 h charging time every 4–5 days) for 10–15 days on their wrist (humans) or collars (dogs). The dog’s monitor was used to collect and store vector magnitude (integrated output) and proximity data (received signal strength indicator, RSSI) [14] in 60-s epochs and sample rate of 30 Hz. The monitors worn by the humans were used solely as beacons; the dog’s monitor (receiver) collected and stored the beacon signal. 

The dogs wore a PetPace Smart-collar for 24 h/day (except 2–3 h charging time every 4–5 days) for 10–15 days. PetPace uses a tri-axial accelerometer to collect activity (vector magnitude) every second. Activity is reported every 2 min as percent of maximal. The 3D-accelerometer data of orientation of the collar is used to identify the body positions. Position is recorded when there is no activity for at least 6 s. PetPace uses acoustic sensors to detect pulse waves. The pulse detection algorithm requires a minimum of 40 recognized good quality beats within 2 min of recording. The beats do not need to be continuous within the 2-min sampling period. Heart rate variability (vasovagal tonal index, VVTI) is determined when pulse rate is collected. VVTI is the natural logarithm of the variance in a minimum of 40 N-N (normal R-R) intervals. Along with short-term VVTI measures, the Health Monitoring System calculates 24-h VVTI (average of all VVTI samples over 24-h period), SDNN (standard deviation of N-N intervals in 24-h period), SDANN (standard deviation of the mean of all 5-min segments of N-N intervals in a 24-h period), SDANN Index (mean of the standard deviations of all the N-N intervals for all 5-min segments), and triangular index. VVTI can be converted to SDNN using the formula SDNN = SQRT(EXP(VVTI). PetPace obtains respiratory rate from evidence of sinus arrhythmia during pulse rate monitoring. 

The foster caretakers were asked to keep daily logs, specifically concerning their interactions with the foster dog (e.g., walking, trimming nails, giving ear medicine, bathing). There were no adverse effects of the collars on the dogs and they all tolerated them without issue. All dogs were walked using a leash attached to a harness; leashes were not attached to the collars.

Actigraph data from the dog’s monitor were downloaded using the Low-Frequency Extension [15] option and screened for wear time using the graphing function in ActiLife v6.13.3 software. Cut-points were set at sedentary (0–1351 cpm), light-moderate (1352–5695 cpm), and vigorous (> 5696 cpm) [1,16] using vector magnitude counts per minute (square root of the sum of squares of each axis of data). Bouts (≥ 10-min duration) were calculated using the scoring function in ActiLife and saved as batch files.

PetPace measures position as sitting, standing, eating/drinking, lying on the back, lying sternally, lying right, lying left, and undefined. For our purposes all lying variables were coded as “0”, sitting as “1”, and standing and eating/drinking as “2.” Undefined positions were removed. When more than one position was determined during a 60-s period, the position with the most seconds/minute was used.

PetPace data (activity, activity level, edited position data, pulse, respiration, VVTI) were merged with Actigraph data (vector magnitude, RSSI) so that all data were lined up per minute of Actigraph activity data throughout the course of the study period (14,400–21,600 min per dog). The resulting data were then separated into sedentary, light-moderate, and vigorous bouts using the bout start and end times generated from the ActiLife batch file. The mean ± SE data for all variables were calculated per bout period.

**Figure 1:**
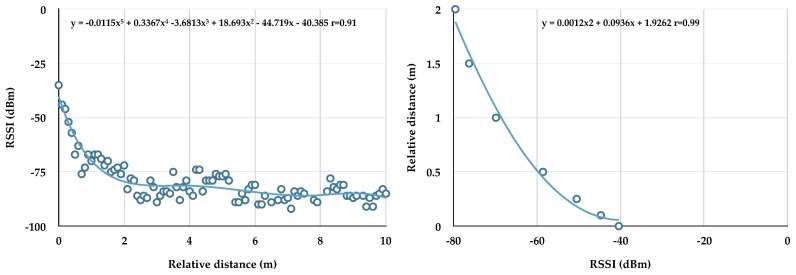
The polynomial relationship between relative distance (meters) vs. received signal strength indicator (RSSI) (**left panel**) and between RSSI vs. calculated distance (**right panel**).

RSSI was converted to relative distance using polynomial equations. Two Actigraph monitors were placed facing each other on a flat surface (floor). One monitor stayed in position while the other was moved at timed intervals by 0.1 m from 0 to 10 m. RSSI (y-axis) was plotted against distance (x-axis) and a 5th order polynomial equation (best fit) applied (Figure 1 left panel). RSSI (y) was calculated from this equation, using meter as x. As relative distance is indistinguishable around ≥2 m, as shown previously [17], a second polynomial equation (2nd order) was generated using 0–2 m (Figure 1 right panel), and this equation used to convert RSSI beacon data collected from the dog’s monitor to distance. Relative distance (0–2 m) measured during sedentary bouts was tested in multiple regression models as a predictor of pulse, respiration, and VVTI. We also examined proximity via signal presence (RSSI between −34 and −91 = 1) and signal absence (no RSSI reading = 0) as described by Clark et al. [7]. Signal presence during sedentary bouts was also tested in multiple regression models as a predictor of pulse, respiration, and VVTI.

Analyses were performed using DataDesk v8.1 software (Data Description, Ithica, NY, USA). Comparisons of pulse, respiration, and VVTI between sedentary and light-moderate bouts and between RSSI absence and RSSI presence were performed using Student’s t-test for paired samples. Comparisons of pulse, respiration, and VVTI between positions (lying vs. sitting vs. standing) were performed using analysis of variance (AVOVA). Pearson correlations were used to examine the relationship between age and bouts, and between Actigraph and PetPace vector magnitude data. Step-wise linear regression models were used to test the effects of Actigraph vector magnitude, position, relative distance (≤ 2 m), and signal presence on pulse, respiration, and VVTI in individual dogs. Statistical significance was set at *p* ≤ 0.05.

## 3. Results

The dogs (toy, mini, and standard American Eskimos) ranged in age between approximately 1 and 12 years. All dogs were altered and of normal weight, and all dogs were healthy except two (lymphoma and multiple cartilaginous exostoses, MCE [18]). Ten of 11 dogs were surrendered by their owners (owner surrender, OS) directly to the rescue group (OS to rescue) or to the shelter (OS to shelter). Those surrendered to the shelter were in the shelter only a few days before being transferred to the rescue group. The physical appearance of Dog 3 indicated he was a stray for a significant amount of time before being secured by animal control. At time of surrender to the shelter, the owner of Dog 11 reported abusive behavior toward the dog in the home. All dogs received medical evaluation and necessary medical care from a Doctor of Veterinary Medicine (DVM) within a few days of coming into the rescue group and before starting in the research study. The dogs and their caretakers were in the study 10–15 days. The dogs spent on average 84% of their time in sedentary behavior, 15% in light-moderate activity, and 1% in vigorous activity (Table 1). Approximate age was positively associated with percent time in sedentary behavior (*r* = 0.79, *p* = 0.01) and inversely related to percent time spent in light-moderate activity (*r* = −0.80, *p* < 0.01) in the healthy dogs (*n* = 9).

To establish the strength of the correlation between activity data collected from the two monitors during bouts, Actigraph activity data (vector magnitude, VM) collected during sedentary, light-moderate, and vigorous bouts were compared to PetPace activity data (VM) (Table 2). Data from each dog showed significant relationships between Actigraph and PetPace activity during sedentary and light-moderate bouts; however, only three dogs had correlations with an *r*-value of 0.7 or greater during sedentary bouts. We therefore used the activity data (VM) generated from the Actigraph monitor, and not the PetPace collar, for all subsequent analyses. Only one dog had enough vigorous bouts to compare the activity data between the two monitors; in this case the activity data were not correlated. 

**Figure 2:**
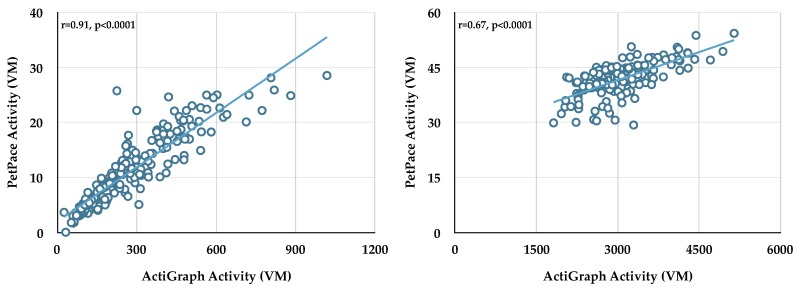
Linear relationship between ActiGraph activity (vector magnitude, VM) and PetPace activity (VM) during sedentary bouts (**left panel**) and light-moderate bouts (**right panel**) in Dog 6.

The raw activity data from Dog 6 (sedentary bouts, left panel; light-moderate bouts, right panel) are shown in Figure 2. Note that while each monitor measures vector magnitude, no standard exists for activity; therefore, each company develops and uses their own scale.

Each bout, whether categorized by ActiLife as sedentary, light-moderate, or vigorous, had corresponding activity data, but not all bouts had pulse, respiration, and VVTI data. For pulse and VVTI, 84 ± 3% of sedentary and 25 ± 6% of light-moderate bouts had corresponding data (sedentary vs. light-moderate, *p* < 0.0001), respectively. For respiration, 81 ± 4% and 17 ± 5% of sedentary and light-moderate bouts had corresponding data (*p* < 0.00001), respectively. The missing values may reflect (a) some bouts between 10–15 min duration may not have had corresponding PetPace data collected, and (b) interfering noises on the PetPace acoustic sensor such as panting.

Table 3 provides the 24-h mean for the number of full days in the study ± SE values for pulse, respiration, and the various HRV time-domain parameters in the 11 dogs. The 24-h mean ± SE for the various HRV calculations in the 11 dogs were as follows: VVTI, 11.6 ± 0.06; SDNN, 332 ± 11; SDANN, 164 ± 13; SDANN index, 291 ± 7. These values are made available for comparison to previous studies that have measured HRV in dogs at rest and in freely active dogs using other methodology (e.g., electrocardiogram).

**Figure 3:**
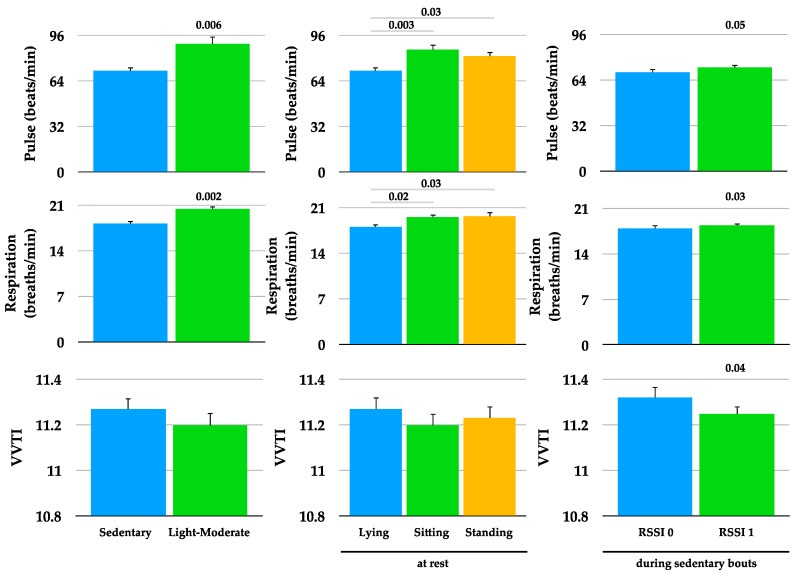
Mean ± SE pulse, respiration, and heart rate variability (VVTI) during sedentary and light-moderate bouts (**left panel**), at rest during lying, sitting, and standing (**middle panel**), and during sedentary bouts in the absence of RSSI signal (RSSI 0) and in the presence of RSSI signal (RSSI 1) (**right panel**). Note for VVTI that y-axis starts at 10.8 (not 0).

To determine whether the dogs’ pulse, respiration, and HRV were affected by activity level and position, data collected during sedentary and light-moderate bouts (left panel), and at rest during lying, sitting, and standing (middle panel), were compared (Figure 3). Pulse and respiration were higher during light-moderate bouts compared to sedentary bouts, and at rest during sitting and standing compared to lying. To determine whether these variables were affected by the absence/presence of the caretaker, data collected during RSSI signal absence (RSSI 0) were compared to RSSI signal presence (RSSI 1) during sedentary bouts (Figure 3). Pulse and respiration were higher, and HRV lower when there was a RSSI signal (RSSI 1, caretaker present) compared to no RSSI signal (RSSI 0, caretaker absent). Neither activity (246 ± 16 vs. 267 ± 11 VM) nor position (0.19 ± 0.05 vs. 0.17 ± 0.02) were significantly different between RSSI 0 and RSSI 1, respectively.

Although we were not able to measure sleep times directly, we did analyze time spent in sedentary bouts (minimum 10 min) exclusively in the lying position (bout could not include sitting or standing). The average time spent in a lying position during RSSI 0 (absence of owner) was 1924 ± 449 min and during RSSI 1 (presence of owner) was 3336 ± 490 min (*p* = 0.07) over the course of the study in the eleven dogs. The average pulse (70 ± 2 vs. 71 ± 2 beats/min), respiration (18 ± 0.5 vs. 18 ± 0.4 breaths/min), and VVTI (11.3 ± 0.04 vs. 11.3 ± 0.04) were not different between RSSI 0 and RSSI 1 while the dogs were in the lying position, respectively.

Using multiple regression models, we examined potential predictors of pulse (Table 4), respiration (Table 5), and VVTI (Table 6) during sedentary bouts in the 11 dogs. The following predictors were included in a step-wise fashion: Actigraph VM (column 4), PetPace position (column 5), relative distance (column 6) (polynomial equation presented in Figure 1, right panel) between the dog and the caretaker, and RSSI presence (column 7). Only one proximity predictor (distance *or* presence of RSSI signal) was added per regression model. Only one gender was added per regression model for dogs that had a male and female caretaker. The direction of the relationship (whether the predictor was positive or negative) is provided for all potential predictors.

Although multiple regression analyses were done only on data collected during sedentary bouts, activity (Actigraph VM) was a positive predictor of pulse (*n* = 10) and respiration (*n* = 9), and a negative predictor of VVTI (*n* = 6). Position was a positive predictor of pulse in three dogs, a positive predictor of respiration in one dog, and a negative predictor of VVTI in two dogs.

Relative distance (0 to 2 m) between the dog and caretaker was a predictor for pulse in six dogs, respiration in five dogs, and VVTI in five dogs. Dog 3 (potential neglect/abuse before coming into rescue) showed increased pulse and respiration the closer he was to his human caretaker. Dogs 8 and 9 showed increased pulse and respiration, and decreased HRV the closer they were to their male caretaker. It is worth noting that dog 9 was receiving daily ear medicine (drops) by the male caretaker throughout the course of the study and the caretaker had written in the log “X does not like it.” Also, dogs 8 and 9 were fostered by the same caretakers simultaneously. Conversely there was no effect on these variables when these dogs were close to their female caretaker. Dog 11 (owner reported abuse in household before surrender to shelter) had increased pulse and respiration, and decreased HRV the closer she was to her male and female caretakers. The two outliers were dogs 6 and 10, who had decreased pulse and increased HRV the closer they were to their caretakers. Interestingly, these two were the only dogs described as “lap dogs” by their caretakers.

RSSI signal presence was a positive predictor for pulse and respiration in four dogs (higher pulse and respiration when caretaker was within signal range). RSSI signal presence was a negative predictor for VVTI in five dogs (lower HRV when the caretaker was within signal range). The one outlier was dog 9 who showed reduced respiration and increased HRV when his male caretaker was within signal range.

## 4. Discussion

The aims of this pilot study were to (a) determine whether combining activity data generated from the Actigraph accelerometer during sedentary and light-moderate bouts with position and physiological data from the PetPace Collar would provide useful and meaningful results in freely moving dogs in a natural environment, and (b) examine the effects of proximity, both relative distance (within a few meters) and RSSI signal presence, between dogs and their caretaker(s) and pulse, respiration, and heart rate variability during sedentary bouts collectively, and on a case-by-case basis. The results suggest that merged data generated from the two monitors will provide a beneficial tool to help determine the effects of proximity, both relative distance (within a few meters) and RSSI signal presence in the field of human-animal interaction research.

Whereas the Actigraph accelerometer has been validated and used in several studies in dogs, limited data are available regarding the PetPace Collar. Other than the published activity data comparison between the Actigraph monitor and PetPace Collar [8], the PetPace Collar pulse algorithm has been compared to standard electrocardiogram and pulse oximetry in 33 pets (cats and dogs) at rest (under anesthesia) [19]. The PetPace Collar has also been shown to be capable of monitoring the general health of working dogs at rest by C. M. Otto, D.V.M., Ph.D., of the Penn Vet Working Dog Center (written communication July 2018).

In the previous study comparing PetPace Collar and Actigraph activity data, a strong relationship (r = 0.92) was found between the activity counts of the two monitors in 10 healthy dogs over seven days [8]. In the current study, we found a weaker relationship between the two monitors (average VM data from 11 dogs: sedentary bouts, *r* = 0.51; light-moderate bouts, *r* = 0.62). The difference in results may be explained by the data analysis methods. Belda et al. compared the sum of activity counts per 60-min periods across seven days whereas we compared the average vector magnitude per activity bout (sedentary and light-moderate) over 10–15 days [8]. The authors determined that 90% of potential PetPace activity data points were obtained during 60-min epochs [8]. In the same study, 76% of potential data points were obtained for pulse and 74% for VVTI [8]. In the current study, we found that 100% of sedentary and light-moderate bouts had corresponding activity data, 84% of sedentary bouts had corresponding pulse and VVTI data, and 25% of light-moderate bouts had corresponding pulse and VVTI data. We also found that 81% of sedentary bouts had corresponding respiration data, and 17% of light-moderate bouts had corresponding respiration data. Both studies demonstrate that the PetPace Collar, as currently designed, is unable to accurately record pulse, respiration, and HRV during vigorous activity. In addition, the PetPace Collar records significantly fewer physiological measures during light-moderate activity compared to sedentary activity.

Twenty-four-hour SDNN, SDANN, and SDANN index values reported in the current study were similar to values published from 24-h ambulatory electrocardiograph recordings in healthy dogs [20,21]. SDNN values in healthy dogs reported in other studies, e.g., [22,23,24], are not comparable to PetPace SDNN values as they were not measured during ambulation and data were collected for 5–60 min vs. 24 h. 

In the current study, pulse and respiration, but not HRV, were significantly higher during light-moderate bouts compared to sedentary bouts and during sitting and standing compared to lying. Similar results were reported by Maros et al. who found differences in heart rate, but not HRV, between walking and sedentary behavior and between lying vs. sitting with no difference between sitting and standing in 14 family dogs [25]. This group also found that HRV was ~70% higher when the dogs were separated from their owners but standing near the experimenter vs. standing by their owner and vs. standing by the experimenter (owner absent) while being petted [25]. They also noted a 70% increase in HRV when the dogs oriented toward a toy vs. sitting. These results led the authors to suggest that HRV could be an indicator of elevated attention, i.e., the dogs were more attentive when the owner was absent (enhanced attention to the missing owner). Others have shown increased HRV under experimental conditions over short periods of time when the dog was separated from their owner [12,13,26]. Our results of higher HRV (and lower pulse and respiration) in the absence vs. presence of the RSSI signal are in agreement with the aforementioned studies, even though the methods were different. Whereas the other studies measured HRV over short periods of time under experimental conditions, we measured HRV over 10–15 days under natural conditions. Zupan et al. suggested that decreased HRV (parasympathetic deactivation) in dogs during a positive stimulus (social reward) was associated with a more positive emotional state [27]. Enhanced attention and/or a less positive emotional state when the caretaker was not within ~10 m (no RSSI signal) may explain higher HRV (and lower pulse and respiration) in the absence vs. presence of the RSSI signal in the dogs in the current study. Further studies to better understand dogs’ HRV responses in the presence and absence of their caretakers in human-animal interaction research are warranted.

We explored relative distance (within ~2 m) during sedentary bouts as a potential predictor of pulse, respiration, and HRV in the individual dogs. Relative distance measures the response of the dog while they are close to their caretaker, from being side-by-side up to 2 m away. We suggest that measuring the dog’s response to relative distance is different than measuring the dog’s response in the absence/presence of the caretaker as discussed in the previous paragraph. The dogs that showed increased pulse and respiration and reduced HRV when closest to their caretaker included the dogs in whom abusive behavior in their previous homes was suspected/reported and a dog receiving daily ear medicine. These results suggest the dogs were least content (more stressed) when side-by-side with their caretakers, and more content (less stressed) when near (a few feet away), but not too close to their caretakers. The two dogs described as lap dogs had the opposite response, indicating, perhaps, that they were most content when closest to their caretakers. Two dogs had gender-specific responses and one dog responded similarly to both her male and female caretaker. These results contradict the theory that higher HRV is associated with enhanced attention and/or a less positive emotional state and more closely support the theory that higher HRV is associated with lower stress, at least when measuring relative distance to within a few meters. The apparent discrepancy in results was likely due to differences in the proximity measure, i.e., absence/presence of caretaker vs. closeness between 0–2 m. We suggest that relative distance (closeness) between the caretaker and the dog may more accurately reflect stress responses, whereas absence/presence of the caretaker reflects attentiveness and/or emotional state in the dog.

We also considered RSSI signal presence (within ~10 m) as a potential predictor of pulse, respiration, and HRV in the individual dogs. RSSI signal presence/absence is presumably measuring the response of the dog while the caretaker is in the home vs. away from the home. Four dogs had higher pulse and respiration and five had lower HRV when there was an RSSI signal, in agreement with results from the mean RSSI data (RSSI 0 vs. RSSI 1) from all 11 dogs. There was one outlier (the dog receiving daily ear drops) who showed the opposite effects and the effects were specific to the male caretaker. It is possible that this dog was most stressed while receiving ear medicine (HRV as marker of stress during relative distance) yet in a more positive emotional state while the caretaker was in the home vs. away from the home.

Multiple regression analysis showed that activity was a predictor, and hence a potential confounding variable, for pulse (*n* = 9 dogs), respiration (*n* = 8 dogs), and heart rate variability (*n* = 5 dogs) during sedentary bouts in the current study. Therefore, activity should be considered when examining physiological responses in freely moving dogs using the PetPace Collar in future studies. 

Limitations of this pilot study include data collected on a small number of dogs and caretakers. Another limitation is that the rescue group and shelters were not provided sufficient information from the owners to best understand the environment the dog lived in before being surrendered, e.g., whether there was a healthy or unhealthy relationship between the dog and caretaker(s). An additional limitation is that we were not able to distinguish whether the dogs were awake or asleep while in the lying position. This is an important point, as Varga et al. showed higher heart rate and lower HRV during wakefulness compared to all sleep states in 12 family dogs [26]. Although it is not proof that the dogs in the current study were sleeping, they did spend, on average, 1.7-fold more time in the lying position when the caretaker was within 10 m (RSSI 1) than when the caretaker was outside of that range (RSSI 0), and pulse, respiration, and HRV were not different between RSSI 1 and RSSI 0 while dogs were in the lying position. Strengths of this study include the collection of activity, proximity, position, and physiology data during sedentary and light-moderate bouts over 10–15 days for 24-h daily under natural conditions in a home environment. 

Future potential applications of using the combined Actigraph Link and PetPace data are virtually limitless and include research to better understand relationships between heart rate variability and sleep [26], emotional states [12,27], attachment behavior [13], shelter assessment [28], animal-assisted therapy [29,30], anxiety-related behavior problems [31], and canine aggression [32] in freely active dogs under natural conditions.

## 5. Conclusions

Our results illustrate the value of combining activity and proximity data generated from the Actigraph accelerometer during sedentary and light-moderate bouts and position and health-related data from the PetPace Collar for monitoring freely active dogs in a natural (home) environment. We demonstrate that the merged data were effective in establishing the effects of proximity, both relative distance and RSSI signal presence, between dogs and their caretaker(s) and pulse, respiration, and heart rate variability during sedentary bouts collectively and on a case-by-case basis. Further research is necessary to determine whether and under what conditions heart rate variability can be used in conjunction with proximity tagging as a marker of stress and/or attention and/or emotional state in companion dogs. 

## Figures and Tables

**Table 1:** Dog characteristics including average sedentary behavior, light-moderate activity, and vigorous activity bout data.

Dog	Age (years)	Weight (kg)	Gender	Health Status	Rescue Status	Days in Study	% Sedentary	% Light- Moderate	% Vigorous
1	~10	10.9	MN	cancer	OS to rescue	10	83	17	0
2	~12	14.5	MN	healthy	OS to rescue	15	91	9	0
3	~4	12.7	MN	healthy	Stray to shelter	12	84	15	1
4	1.5	9.1	MN	MCE	OS to rescue	15	86	13	1
5	~2	7.7	FS	healthy	OS to shelter	14	68	25	7
6	~1	2.7	FS	healthy	OS to rescue	11	77	21	2
7	~8	13.6	FS	healthy	OS to rescue	10	86	13	1
8	~8	12.7	FS	healthy	OS to rescue	10	86	13	1
9	~10	9.1	MN	healthy	OS to shelter	10	83	16	1
10	~10	11.8	FS	healthy	OS to shelter	14	88	12	0
11	~7	11.4	FS	healthy	OS to shelter	12	89	10	1

MN, male neutered; FS, female spayed; OS, owner surrender.

**Table 2:** Number of days in study, number of bouts and average time spent per bout, and relationships between ActiGraph activity data (vector magnitude, AG VM) and PetPace data (vector magnitude, PP VM) during sedentary, light-moderate, and vigorous bouts.

	Sedentary Bouts			Light-Moderate Bouts			Vigorous Bouts		
Dog ID	# of Bouts	Time (min) x ± SE	AG VM v. PP VM (*r*), (*p*)	# of bouts	Time (min) x ± SE	AG VM v. PP VM (*r*), (*p*)	# of bouts	Time (min) x ± SE	AG VM v. PP VM (*r*), (*p*)
1	177	56 ± 7	0.27, =0.0003	93	18 ± 1	0.77, <0.0001			
2	349	49 ± 2	0.31, <0.0001	56	17 ± 1	0.57, <0.0001			
3	244	49 ± 4	0.39, <0.0001	95	15 ± 1	0.62, <0.0001			
4	260	60 ± 4	0.45, <0.0001	92	14 ± 0.5	0.70, <0.0001			
5	196	58 ± 5	0.40, <0.0001	218	16 ± 0.5	0.43, <0.0001	33	13 ± 0.5	0.09, ns
6	216	46 ± 3	0.91, <0.0001	147	17 ± 1	0.67, <0.0001	2	13 ± 1	----------
7	228	48 ± 4	0.37, <0.0001	51	16 ± 1	0.38, =0.006			
8	268	41 ± 3	0.37, <0.0001	41	17 ± 1	0.42, =0.006			
9	242	43 ± 3	0.28, <0.0001	76	15 ± 0.5	0.83, <0.0001			
10	258	57 ± 4	0.95, <0.0001	64	14 ± 1	0.58, <0.0001			
11	169	77 ± 8	0.90, <0.0001	53	14 ± 1	0.80, <0.0001			

ns, non-significant.

**Table 3:** Mean, maximum and minimum ± SE 24-h pulse and respiration, and mean ± SE HRV time-domain parameters.

	Pulse (beats/min)			Respiration (breaths/min)			Heart Rate Variability			
Dog ID	Average	Max	Min	Average	Max	Min	VVTI	SDNN (ms)	SDANN (ms)	SDANN Index (ms)
1	77 ± 2	176 ± 13	39 ± 2	19 ± 0.2	26 ± 0.9	13 ± 0.6	11.7 ± 0.04	351 ± 6	194 ± 13	303 ± 7
2	66 ± 1	163 ± 12	39 ± 1	18 ± 0.2	29 ± 1.3	11 ± 0.3	11.3 ± 0.06	297 ± 10	171 ± 8	243 ± 5
3	67 ± 0.4	129 ± 10	46 ± 2	16 ± 0.2	25 ± 0.6	10 ± 0.2	11.4 ± 0.04	297 ± 5	130 ± 8	273 ± 3
4	74 ± 1	128 ± 4	42 ± 2	19 ± 0	26 ± 0.3	12 ± 0.3	11.6 ± 0.03	327 ± 5	134 ± 16	300 ± 2
5	62 ± 0.6	80 ± 2	45 ± 2	18 ± 0.2	22 ± 0.3	13 ± 0.4	11.6 ± 0.04	333 ± 6	126 ± 7	317 ± 4
6	75 ± 1	156 ± 16	45 ± 4	19 ± 0.2	29 ± 0.6	12 ± 0.6	11.4 ± 0.04	296 ± 6	129 ± 14	270 ± 5
7	79 ± 3	148 ± 10	48 ± 3	19 ± 0.4	25 ± 0.6	13 ± 0.3	11.6 ± 0.03	326 ± 6	145 ± 10	296 ± 3
8	70 ± 1	102 ± 6	48 ± 2	19 ± 0.2	26 ± 0.7	13 ± 0.5	11.5 ± 0.03	311 ± 6	120 ± 5	293 ± 6
9	71 ± 1	134 ± 14	35 ± 2	18 ± 0.2	25 ± 0.6	11 ± 0.6	11.7 ± 0.04	345 ± 6	199 ± 18	301 ± 3
10	67 ± 1	148 ± 11	32 ± 3	17 ± 0.3	27 ± 0.7	10 ± 0.2	11.6 ± 0.06	338 ± 10	202 ± 21	282 ± 3
11	59 ± 4	204 ± 3	35 ± 0.4	15 ± 0.3	26 ± 1	10 ± 0.2	12.1 ± 0.05	430 ± 11	260 ± 20	326 ± 13

**Table 4:** Predictors of pulse during sedentary bouts.

Dog ID	Caretaker Gender	Pulse				
		(*r*)	VM (*p*)	Position (*p*)	Distance (*p*)	Signal Presence (*p*)
1	F	0.45	<0.0001 (+)	ns	ns	ns
2	F	0.42	<0.0001(+)	ns	ns	<0.05 (+)
3	F	0.24	ns	ns	=0.003 (−)	ns
4	F	0.47	<0.0001(+)	ns	ns	ns
5	F	0.46	<0.0001(+)	ns	ns	<0.0001 (+)
6	F	0.38	<0.0001(+)	ns	=0.01 (+)	ns
7	M	-----	ns	ns	ns	ns
	F	0.24	ns	ns	ns	=0.002 (+)
8	M	0.41	=0.0003 (+)	ns	=0.0005 (−)	ns
	F	0.42	=0.0002 (+)	ns	ns	<0.0001 (+)
9	M	0.52	<0.0001(+)	<0.0001 (+)	<0.05 (−)	ns
	F	0.50	<0.0001(+)	<0.0001(+)	ns	ns
10	F	0.36	=0.002 (+)	ns	=0.002 (+)	ns
11	M	0.43	<0.0001(+)	ns	=0.009 (−)	ns
	F	0.43	=0.03 (+)	0.006 (+)	=0.001 (−)	ns

F, female; M, male; (+), positive predictor; (−), negative predictor; ns, non-significant.

**Table 5:** Predictors of respiration during sedentary bouts.

Dog ID	Caretaker Gender	Respiration				
		(*r*)	VM (*p*)	Position (*p*)	Distance (*p*)	Signal Presence (*p*)
1	F	0.20	<0.0001(+)	ns	ns	ns
2	F	0.51	<0.0001(+)	ns	ns	=0.04 (+)
3	F	0.32	<0.05 (+)	ns	=0.002 (−)	ns
4	F	0.28	<0.0001(+)	ns	ns	ns
5	F	0.26	ns	ns	ns	=0.002 (+)
6	F	0.27	=0.0002 (+)	ns	ns	ns
7	M	-----	ns	ns	ns	ns
	F	0.26	ns	ns	ns	=0.002 (+)
8	M	0.36	=0.0002 (+)	ns	=0.03 (−)	ns
	F	0.36	<0.0001(+)	ns	ns	=0.03 (+)
9	M	0.37	ns	ns	=0.007 (−)	na
	M	0.36	<0.0001(+)	ns	na	=0.01 (−)
	F	0.32	=0.001(+)	ns	ns	ns
10	F	0.41	<0.0001(+)	ns	=0.04 (+)	ns
11	M	0.38	ns	ns	<0.0001(−)	ns
	F	0.43	ns	<0.0001(+)	=0.008 (−)	ns

F, female; M, male; (+), positive predictor; (−), negative predictor; ns, non-significant; na, not applicable.

**Table 6:** Predictors of VVTI during sedentary bouts.

Dog ID	Caretaker Gender	VVTI				
		(*r*)	VM (*p*)	Position (*p*)	Distance (*p*)	Signal Presence (*p*)
1	F	0.40	ns	=0.02 (−)	ns	ns
2	F	-----	ns	ns	ns	ns
3	F	-----	ns	ns	ns	ns
4	F	0.41	<0.0001 (−)	ns	ns	=0.05 (−)
5	F	0.35	=0.006 (−)	ns	ns	=0.0003 (−)
6	F	0.15	ns	ns	< 0.05 (−)	ns
7	M	-----	ns	ns	ns	ns
	F	-----	ns	ns	ns	ns
8	M	0.29	ns	ns	< 0.0001 (+)	ns
	F	0.28	ns	ns	ns	<0.0001 (−)
9	M	0.35	=0.01 (−)	ns	=0.03 (+)	na
	M	0.35	=0.0002 (−)	ns	na	=0.0006 (+)
	F	0.33	=0.003 (−)	ns	ns	=0.008 (−)
10	F	0.30	=0.007 (−)	ns	=0.02 (−)	ns
11	M	0.37	=0.03 (−)	ns	=0.0004 (+)	ns
	F	0.42	=0.007 (−)	0.04 (−)	=0.002 (+)	na
		0.40	<0.0001 (−)	ns	na	=0.01 (-)

F, female; M, male; (+), positive predictor; (−), negative predictor; ns, non-significant; na, not applicable.

## References

[1] Yam P.S., Penpraze V., Young D., Todd M.S., Cloney A.D., Houston-Callaghan K.A., Reilly J.J. (2011). Validity, practical utility and reliability of Actigraph accelerometry for the measurement of habitual physical activity in dogs. J. Small Anim. Pract..

[2] Morrison R., Penpraze V., Beber A., Reilly J.J., Yam P.S. (2013). Associations between obesity and physical activity in dogs: A preliminary investigation. J. Small Anim. Pract..

[3] Morrison R., Reilly J.J., Penpraze V., Pendlebury E., Yam P.S. (2014). A 6-month observational study of changes in objectively measured physical activity during weight loss in dogs. J. Small Anim. Pract..

[4] Helm J., McBrearty A., Fontaine S., Morrison R., Yam P. (2016). Use of accelerometry to investigate physical activity in dogs receiving chemotherapy. J. Small Anim. Pract..

[5] Jones S., Dowling-Guyer S., Patronek G.J., Marder A.R., Segurson D’Arpino S., McCobb E. (2014). Use of accelerometers to measure stress levels in shelter dogs. J. Appl. Anim. Welf. Sci..

[6] den Uijl I., Gomez Alvarez C.B., Bartram D., Dror Y., Holland R., Cook A. (2017). External validation of a collar-mounted triaxial accelerometer for second-by-second monitoring of eight behavioural states in dogs. PLoS ONE.

[7] Clark B.K., Winkler E.A., Brakenridge C.L., Trost S.G., Healy G.N. (2018). Using Bluetooth proximity sensing to determine where office workers spend time at work. PLoS ONE.

[8] Belda B., Enomoto M., Case B.C., Lascelles B.D.X. (2018). Initial evaluation of PetPace activity monitor. Vet. J..

[9] Motooka M., Koike H., Yokoyama T., Kennedy N.L. (2006). Effect of dog-walking on autonomic nervous activity in senior citizens. Med. J. Aust..

[10] Kim H.G., Cheon E.J., Bai D.S., Lee Y.H., Koo B.H. (2018). Stress and heart rate variability: A meta-analysis and review of the literature. Psychiatry Investig..

[11] Bogucki S., Noszczyk-Nowak A. (2017). Short-term heart rate variability in dogs with sick sinus syndrome or chronic mitral valve disease as compared to healthy controls. Pol. J. Vet. Sci..

[12] Katayama M., Kubo T., Mogi K., Ikeda K., Nagasawa M., Kikusui T. (2016). Heart rate variability predicts the emotional state in dogs. Behav. Process..

[13] Gacsi M., Maros K., Sernkvist S., Farago T., Miklosi A. (2013). Human analogue safe haven effect of the owner: Behavioural and heart rate response to stressful social stimuli in dogs. PLoS ONE.

[14] Gao V. Proximity and RSSI. https://blog.bluetooth.com/proximity-and-rssi.

[15] Feito Y., Garner H.R., Bassett D.R. (2015). Evaluation of ActiGraph’s low-frequency filter in laboratory and free-living environments. Med. Sci. Sports Exerc..

[16] Morrison R., Penpraze V., Greening R., Underwood T., Reilly J.J., Yam P.S. (2014). Correlates of objectively measured physical activity in dogs. Vet. J..

[17] Dong Q., Dargie W. Evaluation of the reliability of RSSI for indoor localization. Proceedings of the 2012 International Conference on Wireless Communications in Underground and Confined Areas.

[18] Doige C.E. (1987). Multiple cartilaginous exostoses in dogs. Vet. Pathol..

[19] Scheinowitz M., Lascelles B.D.X. (2018). Validation of the Accuracy of PetPace Pulse Rate Measurements (White Paper).

[20] Blake R.R., Shaw D.J., Culshaw G.J., Martinez-Pereira Y. (2018). Poincare plots as a measure of heart rate variability in healthy dogs. J. Vet. Cardiol..

[21] Santos T.H.Y., Araujo M.M.G.d., Gonçalves R.D.S., Sudano M.J., Machado L.H.A., Carvalho L.R., Lourenco M.L.G. (2014). Heart rate variability indices in the time domain in healthy dogs supplemented with omega n-3. Semina: Ciencias Agrárias.

[22] Essner A., Sjöström R., Gustås P., Edge-Hughes L., Zetterberg L., Hellström K. (2015). Validity and reliability properties of canine short-term heart rate variability measures—A pilot study. J. Vet. Behav. Clin. Appl. Res..

[23] Bogucki S., Noszczyk-Nowak A. (2015). Short-term heart rate variability (HRV) in healthy dogs. Pol. J. Vet. Sci..

[24] Jonckheer-Sheehy V.S.M., Vinke C.M., Ortolani A. (2012). Validation of a Polar^®^ human heart rate monitor for measuring heart rate and heart rate variability in adult dogs under stationary conditions. J. Vet. Behav. Clin. Appl. Res..

[25] Maros K., Doka A., Miklosi A. (2008). Behavioural correlation of heart rate changes in family dogs. Appl. Anim. Behav. Sci..

[26] Varga B., Gergely A., Galambos A., Kis A. (2018). Heart Rate and Heart Rate Variability during Sleep in Family Dogs (*Canis familiaris*). Moderate Effect of Pre-Sleep Emotions. Animals.

[27] Zupan M., Buskas J., Altimiras J., Keeling L.J. (2016). Assessing positive emotional states in dogs using heart rate and heart rate variability. Physiol. Behav..

[28] Bergamasco L., Osella M.C., Savarino P., Larosa G., Ozella L., Manassero M., Badino P., Odore R., Barbero R., Re G. (2010). Heart rate variability and saliva cortisol assessment in shelter dog: Human–animal interaction effects. Appl. Anim. Behav. Sci..

[29] Palestrini C., Calcaterra V., Cannas S., Talamonti Z., Papotti F., Buttram D., Pelizzo G. (2017). Stress level evaluation in a dog during animal-assisted therapy in pediatric surgery. J. Vet. Behav..

[30] Melco A.L., Goldman L., Fine A.H., Peralta J.M. (2018). Investigation of Physiological and Behavioral Responses in Dogs Participating in Animal-Assisted Therapy with Children Diagnosed with Attention-Deficit Hyperactivity Disorder. J. Appl. Anim. Welf. Sci..

[31] Wormald D., Lawrence A.J., Carter G., Fisher A.D. (2017). Reduced heart rate variability in pet dogs affected by anxiety-related behaviour problems. Physiol. Behav..

[32] Craig L., Meyers-Manor J.E., Anders K., Sutterlin S., Miler H. (2017). The relationship between heart rate variability and canine aggression. Appl. Anim. Behav. Sci..

